# Acquired Resistance of *Mycobacterium tuberculosis* to Bedaquiline

**DOI:** 10.1371/journal.pone.0102135

**Published:** 2014-07-10

**Authors:** Koen Andries, Cristina Villellas, Nele Coeck, Kim Thys, Tom Gevers, Luc Vranckx, Nacer Lounis, Bouke C. de Jong, Anil Koul

**Affiliations:** 1 Department of Infectious Diseases, Janssen Pharmaceutica, Beerse, Belgium; 2 Department of Biomedical Sciences, Institute of Tropical Medicine, Antwerp, Belgium; University of Cambridge, United Kingdom

## Abstract

Bedaquiline (BDQ), an ATP synthase inhibitor, is the first drug to be approved for treatment of multi-drug resistant tuberculosis in decades. *In vitro* resistance to BDQ was previously shown to be due to target-based mutations. Here we report that non-target based resistance to BDQ, and cross-resistance to clofazimine (CFZ), is due to mutations in Rv0678, a transcriptional repressor of the genes encoding the MmpS5-MmpL5 efflux pump. Efflux-based resistance was identified in paired isolates from patients treated with BDQ, as well as in mice, in which it was confirmed to decrease bactericidal efficacy. The efflux inhibitors verapamil and reserpine decreased the minimum inhibitory concentrations of BDQ and CFZ *in vitro*, but verapamil failed to increase the bactericidal effect of BDQ in mice and was unable to reverse efflux-based resistance *in vivo*. Cross-resistance between BDQ and CFZ may have important clinical implications.

## Introduction

BDQ (Sirturo, TMC207) was recently granted accelerated approval as part of combination therapy to treat adults with multi-drug resistant pulmonary tuberculosis (TB) when an effective treatment regimen cannot otherwise be provided [1]. Its mechanism of action, inhibition of ATP synthase, was discovered through whole genome sequencing of resistant mutants that were selected *in vitro* [2]. As BDQ will now be used in clinical practice, it is important to understand any additional, non-target based mechanism(s) that lead to decreased sensitivity or resistance, so that appropriate drug susceptibility testing methods can be developed.

Several target-based resistance mutations in the *atpE* gene have been described previously [2]–[4], but when a Luria-Delbruck fluctuation assay was used to generate a larger set of BDQ-resistant mutants, target-based mutations were identified in only 30% (15/53) of the isolates, suggesting additional mechanism(s) of resistance [3]. Although the mutation rates observed in the above study suggested a genotypic rather than a phenotypic mechanism of resistance, whole genome sequencing of two non-*atpE* mutants initially did not reveal genomic changes.

Here we selected *Mycobacterium tuberculosis* isolates with increased minimal inhibitory concentrations (MICs) for BDQ *in vitro*, in mice and in patients, and studied these for non-target based mechanisms of resistance. After identifying the efflux system MmpS5-MmpL5 as being responsible for non-target based resistance, we studied the effect of efflux pump inhibitors (EPIs) on BDQ’s *in vitro* MICs and *in vivo* efficacy in mice. Hartkoorn *et al.* recently described the *in vitro* isolation of CFZ-resistant strains, with cross-resistance to BDQ, by up-regulation of the MmpS5-MmpL5 efflux system [5]. Both CFZ and BDQ can therefore select for efflux-based resistance, leading to cross-resistance between the two drugs.

## Results

### Non-target based resistance to BDQ is linked to mutations in *Rv0678*

Non-*atpE* mutant strains EH 3.2 and EH3.6 were re-analyzed by 454 sequencing for the presence of mutations and/or insertions. In non-*atpE* mutant strain EH 3.2, a non-synonymous single nucleotide polymorphism was observed in *Rv0678* gene (A202G leading to S68G), while in strain EH 3.6, a non-synonymous single nucleotide polymorphism was observed in *ppsC* gene (G5408T leading to G1803V). The *ppsC* gene is involved in phenolpthiocerol and phthiocerol dimycocerosate biosynthesis [6] and has not been linked to antibiotic resistance, while *Rv0678* gene has been implied in decreased sensitivity to azoles [7] and an investigational compound with antitubercular activity [8]. A focused analysis and search for variants in the candidate gene (*Rv0678* from mutant EH3.2) in mutant EH3.6 revealed an additional inconsistency in the read assembly, reflecting a large insertion at position 272 in *Rv0678* gene. Although the full length sequence of this insertion could not be revealed by the 454 sequencing analysis, the beginning and end of the nucleotide sequence of this insert suggested an insertion of mobile element IS*6110*, resulting in a disruption at the protein level from amino acid 91 onwards. These findings suggested that in both non-*atpE* mutant strains *Rv0678* was a candidate gene responsible for non-target based resistance to BDQ.

**Figure 1:**
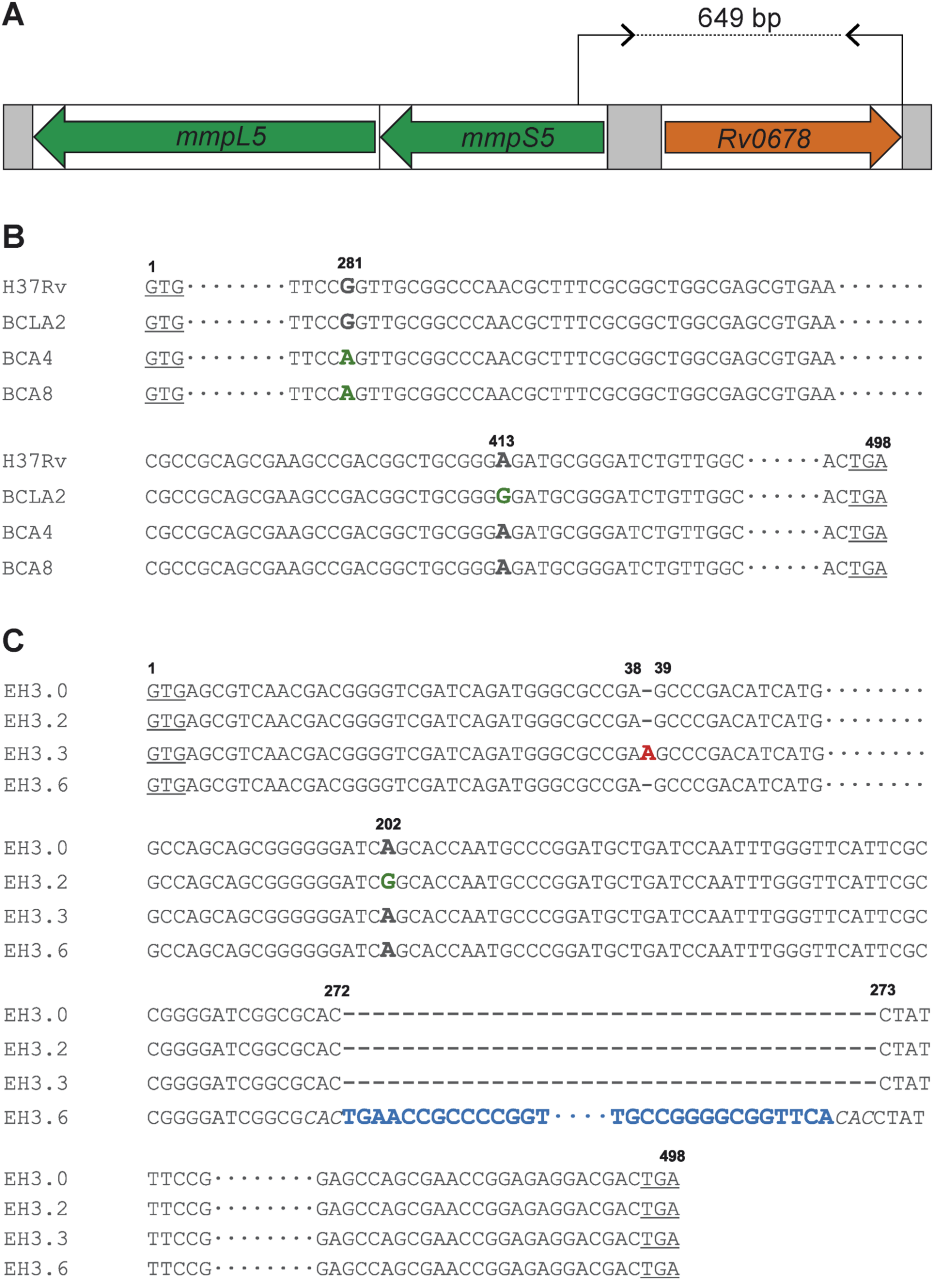
Mutations in *Rv0678* gene of *M. tuberculosis* BDQ resistant strains. **A.** PCR fragment amplified for sequencing and mapping of mutations in *Rv0678* gene of BDQ resistant strains in (**B**) H37Rv-derived mutants and (**C**) EH 3.0-derived mutants. Codons START and STOP of *Rv0678* gene are underlined. The nucleotide positions are indicated on top of each mutation. Mutations are bold and colored: missense mutations are indicated in green, insertions are highlighted in red, and the 1.3 Kb insertion sequence IS*6110* is colored in blue. The direct repeats of IS*6110* are indicated in italics.

To confirm the results of 454 sequencing, a fragment containing *Rv0678* gene and the region 143 bp upstream was Sanger sequenced (Fig. 1). One additional *in vitro* isolate (EH3.3) and 3 isolates from mice that had been treated with combinations including BDQ, CFZ, levofloxacin and amikacin (BCLA2, BCA4 and BCA8) were also included. All additional isolates had genomic changes in *Rv0678*, either a missense mutation (in EH 3.2, BCLA2, BCA4 and BCA8), or a single nucleotide insertion (EH 3.3) or an IS*6110* insertion sequence (EH 3.6) (Fig. 1, Table 1).

**Table 1:** Mutations in *Rv0678* gene and MICs of BDQ of resistant preclinical strains.

		Mutation in Rv0678		
Strain	Description	DNA	Protein	BDQ MIC (µg/ml)
H37Rv	wild type *M. tuberculosis* strain	wt	wt	0.063
BCLA 2	BDQ-R mutant, H37Rv-derived, no *atpE* mutations	A413G	E138G	0.250 (×4)
BCA 4	BDQ-R mutant, H37Rv-derived, no *atpE* mutations	G281A	R94Q	0.500 (×8)
BCA 8	BDQ-R mutant, H37Rv-derived, no *atpE* mutations	G281A	R94Q	0.500 (×8)
BK12	BDQ-R mutant, H37Rv-derived, *atpE* mutation [2]	no mutation	no mutation	2.000 (×32)
LV13	BDQ-R mutant, H37Rv-derived, *atpE* mutation [24]	Ins G 192–193	frameshift	4.000 (×64)
EH 3.0	MDR *M. tuberculosis* strain [3]	wt	wt	0.125
EH 3.2	BDQ-R mutant, EH 3.0-derived, no *atpE* mutations [3]	A202G	S68G	0.500 (×4)
EH 3.6	BDQ-R mutant, EH 3.0-derived, no *atpE* mutations [3]	IS*6110 *nt 272	disruption	1.000 (×8)
EH 3.3	BDQ-R mutant, EH 3.0-derived, no *atpE* mutations [3]	Ins A 38–39	frameshift	1.000 (×8)

The MICs of BDQ-R preclinical strains derived from either the drug susceptible H37Rv or the MDR EH3.0 parent strains are shown. Fold-changes between brackets represent the difference between resistant and wild-type MICs. **wt**: wild-type; **Ins**: insertion; **nt**: nucleotide**; BDQ**: bedaquiline; **BDQ-R**: bedaquiline-resistant.

The clinical relevance of mutations in *Rv0678* gene was studied by sequencing 6 paired samples (baseline and post-baseline) with increased post-baseline MICs for BDQ (2 to 16 fold, see below) from a clinical trial (NCT00540449), in which MDR-TB patients were treated with BDQ and a background MDR-TB regimen. None of the post-baseline isolates had mutations in the ATP synthase operon, while all 6 did have genomic changes in the *Rv0678* gene (Table 2). Three isolates had a missense mutation, and three had changes leading to the disruption of the Rv0678 protein: a single nucleotide deletion, a single nucleotide insertion (both resulting in a frameshift) and a transposon IS*6110* insertion in position 172 of *Rv0678* gene. In some cases, mixed populations of parent and mutant strains were observed, due to the fact that DNA was extracted directly from sputum cultures.

**Table 2:** MICs of BDQ and CFZ of clinical isolates, in the absence and presence of reserpine.

		Mutation in Rv0678		MICs (µg/ml)			
Patient	Isolate	DNA	protein	BDQ	BDQ+RES	CFZ	CFZ+RES
Patient A	Baseline	wt	wt	0.125	0.016 (:8)	1.000	0.125 (:8)
	Post-baseline	T124C	W42R	0.250 (×2)	0.125 (:2)	2.000 (2)	0.250 (:8)
Patient B	Baseline	wt	wt	0.125	0.031 (:4)	0.500	0.125 (:4)
	Post-baseline	A97G	T33A	0.500 (×4)	0.250 (:2)	2.000 (×4)	1.000 (:2)
Patient C	Baseline	wt	wt	0.063	≤0.002 (:>32)	0.500	0.125 (:4)
	Post-baseline	C107T	A36V	0.500 (×8)	0.125 (:4)	4.000 (×8)	1.000 (:4)
Patient D	Baseline	wt	wt	0.063	0.016 (:4)	0.500	0.125 (:4)
	Post-baseline	Del C 212	frameshift	0.500 (×8)	0.250 (:2)	1.000 (×2)	0.250 (:4)
Patient E	Baseline	wt	wt	0.063	0.008 (:8)	0.250	0.063 (:4)
	Post-baseline	Ins IS6110 nt 172	frameshift	0.500 (×8)	0.031 (:16)	1.000 (×4)	0.250 (:4)
Patient F	Baseline	wt	wt	0.016	0.008 (:2)	0.500	0.250 (:2)
	Post-baseline	Ins C 141–142	frameshift	0.250 (×16)	0.125 (:2)	4.000 (×8)	4.000 (:1)

MICs and mutations in *Rv0678* gene are shown for baseline and post-baseline isolates of each patient. For MICs without reserpine, fold-changes between brackets represent the difference between resistant and wild-type MICs. For MICs in the presence of reserpine (3 µg/ml), fold-changes between brackets represent the differences between MIC with and without reserpine. **wt**: wild-type; **Ins**: insertion; **Del**: deletion; **nt**: nucleotide; **BDQ**: bedaquiline; **CFZ**: clofazimine; **RES**: reserpine.

### Cross resistance with CFZ and effect on efflux pump inhibitors on MICs

MICs of BDQ and CFZ of all preclinical and clinical strains, in the absence and presence of the EPIs verapamil and/or reserpine were determined (Tables 2 and 3).

**Table 3:** MICs of BDQ and CFZ of preclinical strains with EPIs.

	MIC (µg/ml)					
Strain	BDQ	BDQ+VER	BDQ+RES	CFZ	CFZ+VER	CFZ+RES
H37Rv	0.063	0.004 (:16)	0.016 (:4)	0.250	0.031 (:8)	0.125 (:2)
BCLA 2	0.250 (×4)	0.031 (:8)	0.125 (:2)	0.500 (×2)	0.125 (:4)	0.500 ( = )
BCA 4	0.500 (×8)	0.125 (:4)	0.250 (:2)	1.000 (×4)	0.500 (:2)	1.000 ( = )
BK12	2.000 (×32)	1.000 (:2)	2.000 ( = )	0.250 ( = )	0.031 (:8)	0.063 (:4)
LV13	4.000 (×64)	2.000 (:2)	4.000 ( = )	1∶000 (×4)	0.500 (:2)	1.000 ( = )
EH 3.0	0.125	0.008 (:16)	0.016 (:8)	0.250	0.016 (:16)	0.031 (:8)
EH 3.2	0.500 (×4)	0.016 (:32)	0.250 (:2)	1.000 (×4)	0.016 (:64)	0.125 (:8)
EH 3.6	1.000 (×8)	0.125 (:4)	0.500 (:2)	2.000 (×8)	1.000 (:2)	1.000 (:2)
EH 3.3	1.000 (×8)	0.250 (:4)	0.500 (:2)	>2.000 (≥×8)	2.000 (≥:1)	2.000 (≥:1)

MICs of BDQ and CFZ of preclinical strains, in the absence and presence of verapamil (40 µg/ml) or reserpine (3 µg/ml). Each value is the average of 3 independent experiments. For MIC without EPIs, fold-changes between brackets represent the difference between resistant and wild-type MICs. For MICs in the presence of EPIs, fold-changes between brackets represent the differences between MIC with and without EPI. **BDQ**: bedaquiline; **CFZ**: clofazimine; **RES**: reserpine; **VER**: verapamil.

In preclinical non-*atpE* mutants (Tables 1 and 3), mutations resulting in a disruption of the protein Rv0678 correlated with the higher BDQ MIC increases (8-fold), whereas the effects of the single amino acid changes were more moderate (4–8 fold). *Rv0678* mutants were cross-resistant to CFZ and MIC increases for BDQ and CFZ were very similar. The EPIs verapamil and reserpine reduced the MICs of both BDQ and CFZ, but this effect decreased in mutants with higher initial MICs. The effect of these EPIs was therefore more pronounced for the wild type strains H37Rv and EH3.0. The EPIs had minor effects on the BDQ MICs of the preclinical *atpE* mutants BK12 and LV13. Isolate LV13 carries a double mutation (*Rv0678* and *atpE*) and is therefore cross-resistant to CFZ.

For clinical isolates (Table 2), the increases in MIC ranged from 2-fold to 16-fold, depending on the mutation. Isolates with 8 and 16-fold MIC increases had a disrupted *Rv0678* gene. *Rv0678* mutants were cross-resistant to CFZ and MIC increases for BDQ and CFZ were again very similar. The EPI reserpine used at 3 µg/ml reduced the MICs of both BDQ and CFZ, but efflux pumps were less susceptible to reserpine when the MIC levels were high, resulting in smaller MIC decreases. Concentrations of reserpine greater than 3 µg/ml inhibited the growth of some clinical isolates. Some clinical strains were also tested with verapamil with similar MIC decreases for BDQ and CFZ (results not shown).

### *Rv0678* mutations lead to upregulation of MmpS5-MmpL5 protein expression

We performed whole mycobacterial quantitative proteome analysis comparing mutants EH3.2 and EH3.6 to the wild-type isogenic strain, EH 3.0. Using iTRAQ-labeling and a mass spectrometric (LC-MS/MS) approach, we identified from each sample approximately 2000 proteins of which 1455 could be quantified in terms of relative protein expression (Table S1). A small subset of 25 proteins was found to be deregulated in mutants versus wild-type strain. We regarded proteins showing an increase or decrease with a fold change >1.3 as deregulated. The top upregulated and downregulated proteins are shown in Table 4 and Table S2, respectively. In both EH3.2 and EH3.6 mutant strains, the highest upregulated protein identified was MmpS5 (mycobacterial membrane protein small, Rv0677c), an outer membrane protein known to be involved in multi-drug transport [7]. MmpS5 expression was more than 18-fold and 4-fold upregulated in mutant strains EH3.6 and EH3.2 respectively, as compared to parent strain EH3.0. MmpS5 along with MmpL5, a transmembrane channel, forms the MmpS5-MmpL5 efflux pump complex, belonging to Resistance Nodulation Cell Division (RND) family of mycobacterial transporters [9]. Four out of the 13 *mmpL* genes of *M. tuberculosis* are transcriptionally coupled with *mmpS* genes, and usually function as a complex to transport out different substrates. In both mutant strains, strong upregulation of MmpL5 protein was observed, about 6- and 2-fold in EH3.6 and EH3.2 respectively (Table 4). Other prominent differentially regulated proteins were Rv0559c and Rv0455c, whose function is not known as yet and belong to conserved hypothetical proteins family. *Rv0559c* gene is known to be in operon with *Rv0560c*, which was not found among the quantifiable proteins in our dataset.

**Table 4:** Top 10 upregulated proteins of the BDQ-resistant strains EH3.6 and EH 3.2 compared to EH3.0.

Rv number	gene	Description	Fold changeEH3.6/EH3.0	Fold change EH3.2/EH3.0
Rv0677c	*mmpS5*	Possible conserved membrane protein MmpS5	18.7	4.6
Rv0676c	*mmpL5*	Probable conserved transmembrane transport protein MmpL5	5.9	2.3
Rv0559c	*Rv0559c*	Possible conserved secreted protein	5.5	1.9
Rv0455c	*Rv0455c*	Conserved protein	3.3	1.4
Rv2936	*ddrA*	Daunorubicin-dim-transport ATP-binding protein ABC transporter DrrA	1.8	2.0
Rv2610c	*pimA*	Alpha-mannosyltransferase PimA	1.8	1.7
Rv1426c	*lipO*	Probable esterase LipO	1.8	1.4
Rv0064A	*vapB1*	Possible antitoxin VapB1	1.7	1.7
Rv3836	*Rv3836*	Conserved hypothetical protein	1.7	2.1
Rv0736	*rslA*	Anti-sigma factor RslA	1.7	1.5

Proteins displaying the highest differential upregulation factors (top 10 proteins) are indicated. The descriptions of the genes were retrieved from TubercuList (http://tuberculist.epfl.ch/).

The *M. tuberculosis* genome contains several efflux pump genes belonging to 5 families of transporters namely, ATP-Binding Cassette (ABC) superfamily, Major Facilitator Superfamily (MFS), RND family, Multidrug and Toxic Compound Extrusion (MATE) family and Small Multidrug Resistance (SMR) family. We further analyzed the expression of other efflux pump proteins that could be quantified in our whole mycobacteria proteome dataset. From this dataset, we could identify 13 proteins belonging to different efflux pump families (Table 5) and the only other protein which was slightly overexpressed in the mutant strains was DrrA (Daunorubicin-dim-transport resistance ATP-binding protein), an ABC transporter probably involved in daunorubicin resistance and phthiocerol dimycocerosate transport [6].

**Table 5:** Regulation of efflux pumps proteins in EH3.6 and EH3.2.

Rv number	Gene	Efflux Pump Family	Description	Fold changeEH3.6/EH3.0	Fold change EH3.2/EH3.0
Rv0676c	*mmpL5*	RND	Probable conserved transmembrane transportprotein MmpL5	5.9	2.3
Rv1183	*mmpL10*	RND	Probable conserved transmembrane transportprotein MmpL10	1.0	0.8
Rv0206c	*mmpL3*	RND	Possible conserved transmembrane transportprotein MmpL3	1.0	1.0
Rv1877	*Rv1877*	MFS	Probable conserved integralmembrane protein	1.2	1.1
Rv0341	*iniB*	Membrane protein	Isoniazid inductible geneprotein IniB	1.0	1.4
Rv0342	*iniA*	Membrane protein	Isoniazid inductible geneprotein IniA	1.0	1.3
Rv2936	*drrA*	ABC	Daunorubicin-dim-transport ATP-bindingprotein ABC transporter DrrA	1.8	2.0
Rv1747	*Rv1747*	ABC	Probable conserved transmembraneATP-binding protein ABC transporter	1.1	1.1
Rv1463	*Rv1463*	ABC	Probable conserved ATP-bindingprotein ABC transporter	1.1	1.0
Rv1458c	*Rv1458c*	ABC	Probable unidentified antibiotic-transportATP-binding protein ABC transporter	1.0	1.0
Rv2477c	*Rv2477c*	ABC	Probable macrolide-transportATP-binding protein ABC transporter	1.0	1.0
Rv2688c	*Rv2688c*	ABC	Antibiotic-transport ATP-binding protein ABC transporter	0.9	1.0
Rv1218c	*Rv1218c*	ABC	Probable tetronasin-transport ATP-bindingprotein ABC transporter	0.6	1.0

Efflux pump proteins were selected from the complete dataset of the proteome analysis (Table S1). The descriptions of the genes were retrieved from TubercuList (http://tuberculist.epfl.ch/).

### Overexpression strain of MmpS5-MmpL5 has increased BDQ and CFZ MICs

In order to confirm that overexpression of the MmpS5-MmpL5 efflux pump confers resistance to BDQ and CFZ, 3 strains overexpressing either MmpS5, MmpL5 or MmpS5-MmpL5 were generated. For this, plasmids derived from the replicative vector pSD5 [10] (Fig. S2), containing the corresponding genes under control of the strong promoter P_Hsp65_ were transformed in the wild type strain *M. tuberculosis* H37Rv. The MICs of BDQ and CFZ of MmpS5-MmpL5 overexpression strain were increased by 4-fold and 2-fold respectively when compared to the control strain H37Rv pSD5 (Table 6). In the presence of verapamil, the MIC levels of BDQ and CFZ decreased by 4-fold and 2-fold respectively. When MmpS5 and MmpL5 were overexpressed separately, no increase in the MIC was observed (Table 6). These results suggest that BDQ and CFZ are substrates of the efflux pump, and that the entire operon MmpS5-MmpL5 must be overexpressed in order to transport these drugs.

**Table 6:** MICs of MmpS5 and MmpL5 overexpression strains.

		MIC (µg/ml)			
Strain::plasmid	Description	BDQ	BDQ+VER	CFZ	CFZ+VER
H37Rv	wild type *M. tuberculosis* strain	0.063	0.008	0.250	0.063
H37Rv::pSD5	H37Rv strain containing the control plasmid pSD5	0.063	0.004	0.250	0.031
H37Rv::pCVGA30	H37Rv-derived strain overexpressing MmpS5-MmpL5	0.250	0.063	0.500	0.250
H37Rv::pCVGA26	H37Rv-derived strain overexpressing MmpS5	0.063	0.004	0.250	0.031
H37Rv::pCVGA28	H37Rv-derived strain overexpressing MmpL5	0.063	0.008	0.250	0.063

MICs of strains overexpressing MmpS5, MmpL5 or the operon MmpS5-MmpL5 in the absence and presence of verapamil (40 µg/ml) are indicated. Each value is the average of at least 2 independent experiments. **BDQ**: bedaquiline; **CFZ**: clofazimine; **VER**: verapamil.

### No fitness cost associated with mutations in *Rv0678* gene

To assess the effect of *Rv0678* mutations on fitness *in vitro*, 2 independent 7H9 broth cultures were inoculated with a mixture [10^4^ colony forming units (CFU)/ml each] of the isogenic H37Rv strain and a BDQ resistant mouse isolate (BCLA2 or BCA4). Samples were taken after 0, 3, 5, 7, 10, 12 and 14 days to determine CFUs. No difference in growth was observed (Fig. S1A). To assess the effect of *Rv0678* mutations on virulence *in vivo*, 8 groups of 8 mice were infected intravenously with either wild-type H37Rv or the BDQ resistant (mouse) isolates BCLA2, BCA4 and BCA8. Groups were sacrificed after one day, 2, 4 and 6 weeks to determine lung CFUs. No differences in growth were observed (Fig. S1B).

### Efflux-based resistance leads to a translational effect which cannot be rescued by verapamil

**Figure 2:**
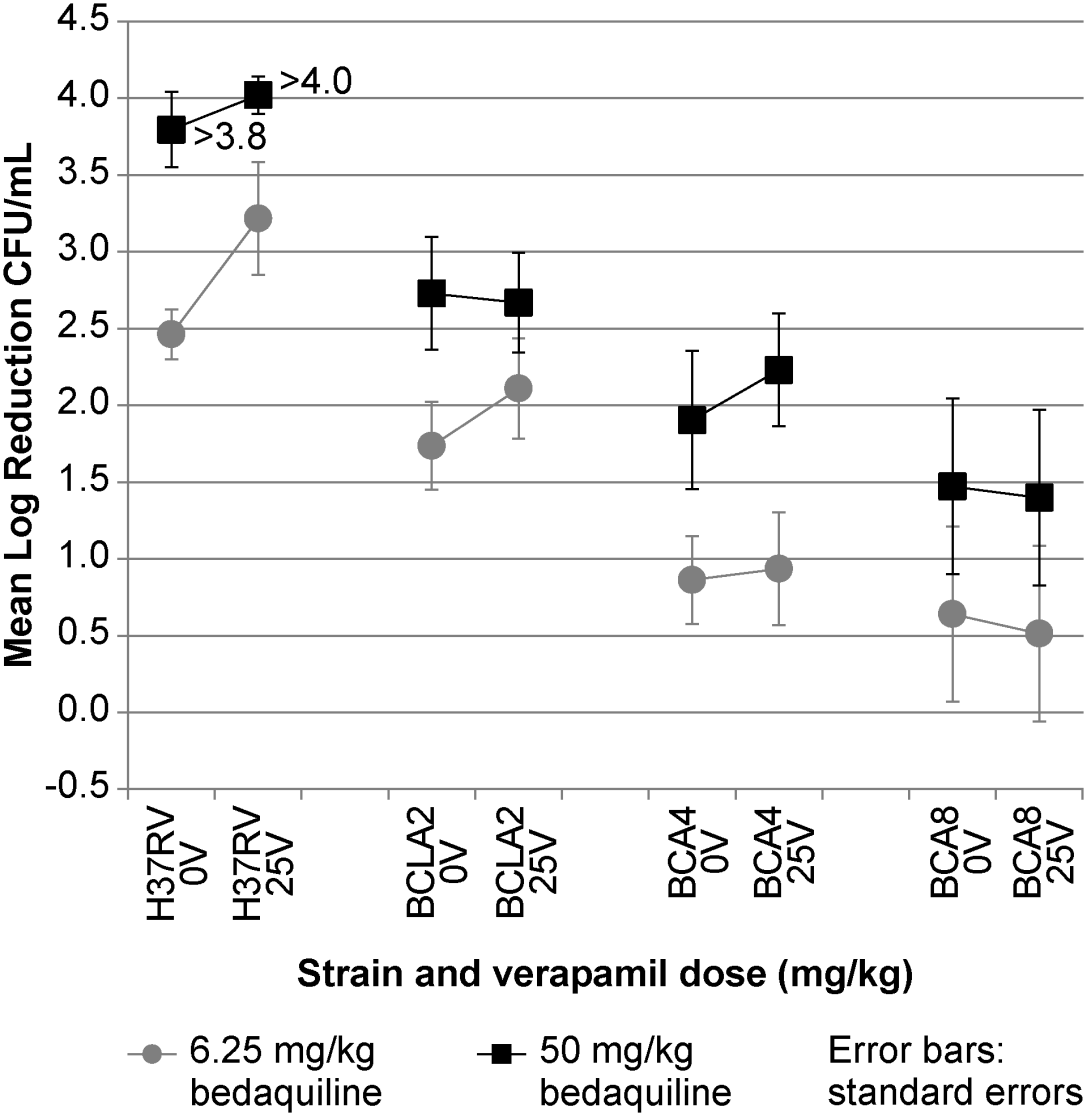
Translational effect of non-target based resistance to BDQ in mice and inability of verapamil to reverse it. Log kill values of wild type H37Rv and *Rv0678* mutants in mice treated with BDQ at 6.25 or 50 mg/kg (5x/week), in the presence (25 V) or absence (0 V) of 25 mg/kg verapamil for 4 weeks. Values represent the median log reduction versus baseline CFU for 6 mice.

To assess the effect of increased MICs on the efficacy of BDQ, groups of 6 mice were infected IV with either wild-type H37Rv or the BDQ resistant (mouse) isolates BCLA2, BCA4 and BCA8. Treatment started at 12 days post infection with 6.25 mg/kg or 50 mg/kg of BDQ, in the absence or presence of verapamil dosed at 25 mg/kg. After 4 weeks of treatment, mice were sacrificed and lung CFU titers determined (Fig. 2). Although BDQ still reduced CFU counts after 4 weeks, all mutants were clearly less susceptible to the bactericidal activity of BDQ compared to the wild type strain H37Rv. The resistance of the least resistant strain BCLA2 (MIC increase 3-fold) could be overcome by increasing the BDQ dose 8-fold (from 6.25 mg/kg to 50 mg/kg), but could not completely overcome resistance of strains BCA4 and BCA8 (MIC increase 8-fold). Adding verapamil to BDQ slightly increased the bactericidal effect after 4 weeks in several groups of mice, but verapamil adjunctive therapy was never significantly better than BDQ monotherapy (p>0.05).

## Discussion

During our search for alternative mechanisms of resistance to BDQ, we observed that non-*atpE* mutants had reduced MICs when tested in the presence of the EPIs reserpine and verapamil. While this observation suggested that efflux could be involved in emergence of resistance, it was subsequently observed by ourselves and others [11] that MICs of wild-type, non-resistant strains were also significantly reduced when tested in the presence of EPIs: MIC levels for BDQ and CFZ indeed decrease 16 -fold in the presence of verapamil [11] (Table 3). An efflux-based mechanism is therefore implied in intrinsic resistance to BDQ, in addition to a possible role in acquired resistance.

A detailed re-analysis of non-*atpE* mutants led to the identification of genomic changes in *Rv0678*, initially in 2 strains with increased MICs, isolated *in vitro*. We subsequently identified mutations in *Rv0678* of all 10 additional strains investigated, including 6 from patients treated with BDQ in a clinical trial.

**Figure 3:**
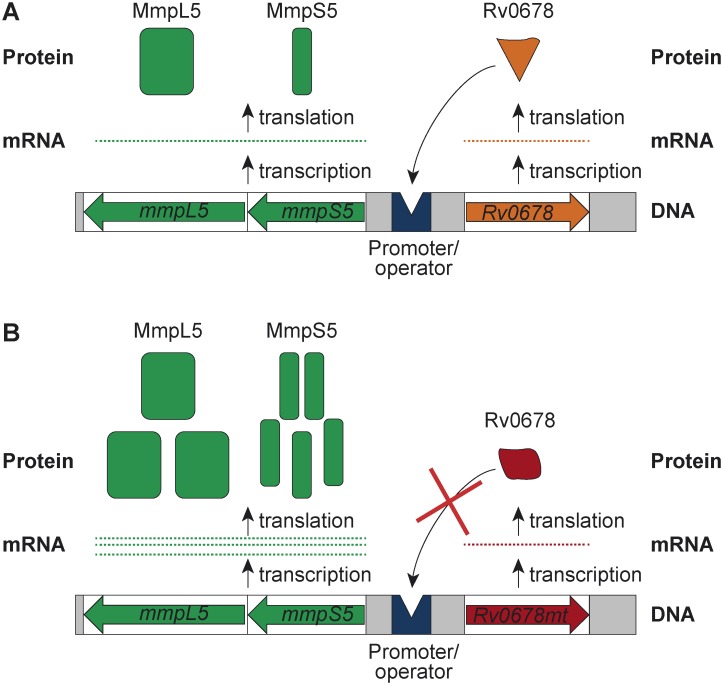
Mechanism of BDQ and CFZ resistance in *Rv0678* mutants. **A.** Regulation of *mmpS5* and *mmpL5* transcription by wild type Rv0678 repressor. Rv0678 protein binds to the intergenic region located between *Rv0678* and *mmpS5*, which contains the-10 consensus boxes of promoters for both *Rv0678* and *mmpS5*. This prevents the RNA polymerase to start transcription, resulting in the decrease of expression of MmpS5, MmpL5 and Rv0678 proteins. In response to an unknown stimulus, the regulator detaches from DNA and transcription can be resumed. **B.** Lack of regulation in *Rv0678* mutants. The strains carrying frame shifting mutations in *Rv0678* will not produce a functional repressor, thus the transcription of *mmpS5, mmpL5* and *Rv0678* will be increased. If the mutation results in an amino acid polymorphism, the protein may still be functional, but with reduced DNA-binding ability depending on the location of the mutation. In either case, the final consequence will be an increase in the expression levels of the proteins MmpS5, MmpL5 and Rv0678.

Drug efflux has recently been highlighted as an important acquired resistance mechanism in *M. tuberculosis* [12]. Mutations and/or insertions in *Rv0678* have previously been described to contribute to resistance to antifungal azoles [7], to an oxazole with anti-tuberculosis activity [8] and to CFZ [5]. The *Rv0678* gene and the *mmpS5-mmpL5* operon are located in opposite orientations in the genome (Fig. 3). The respective-10 consensus boxes of their promoters are placed in a palindromic region where the protein *Rv0678* hypothetically binds, preventing transcription of both the *Rv0678* gene and the *mmpS5-mmpL5* operon [7]. Since *Rv0678* is a negative regulator of MmpS5 and MmpL5, mutations in this gene lead to an increased expression of this efflux pump [7]. All non-*atpE* mutants resistant to BDQ carried a different mutation in *Rv0678* regulator, including missense mutations, single nucleotide insertions, deletions, and IS*6110* insertion sequences. Acquisition of resistance due to IS*6110* insertion was described recently [8], but it has not been reported to disrupt the *Rv0678* gene. Different mutations had different effects on the MICs, presumably because they result in different conformational changes of Rv0678 protein, or in its complete disruption. Frameshift mutations led to the highest increases in MICs (8- or 16-fold), compared to amino acid changes (2 to 8-fold). Proteome data showed that the mutant EH 3.2 (carrying an amino acid substitution) and EH 3.6 (which has an IS*6110* insertion) have MmpS5 and MmpL5 overexpressed. For these strains, the MICs are similar, but the effect of EPIs was less pronounced for the mutant with the disrupted regulator (EH 3.6) and the largest overexpression of MmpS5-MmpL5.

Although we cannot entirely exclude the possibility that additional efflux pumps may be involved in non-target based acquired resistance to BDQ, the fact that mutations in *Rv0678* gene were found in all investigated isolates, together with the proteome data demonstrating overexpression of MmpS5-MmpL5, support the conclusion that the MmpS5-MmpL5 efflux pump is responsible for non-target based acquired resistance. Moreover, the increase in BDQ MIC of the overexpression strain H37Rv pCVGA30 suggests that BDQ is a substrate of MmpL5. This RND pump may also be responsible for the observed intrinsic resistance to BDQ as described above. CFZ is apparently also a substrate of the same efflux pump, as all non-*atpE* strains with increased MICs for BDQ were cross-resistant to CFZ, and the effects of EPIs on the MICs of CFZ and BDQ were very similar.

As efflux inhibition may theoretically increase the bactericidal and sterilizing efficacy of existing drugs in a regimen, the addition of EPIs to TB regimens has the potential to enhance mycobacterial killing *in vivo* [11], [13]. Mouse efficacy data obtained with rifampicin indeed suggest increased bactericidal and sterilizing efficacy of a regimen including verapamil [13]. The excellent potentiating effect of verapamil on the bacteriostatic effect (MIC) of BDQ was very promising, and raised the expectation that the combination of BDQ with verapamil would also result in a significantly better bactericidal effect *in vivo*. In this context, the results shown in figure 2 are disappointing and the reasons for this minor effect on killing in mice need to be further investigated. Different expression levels of efflux pumps *in vivo* versus *in vitro*, a different mechanism of bacteriostatic (MIC) and bactericidal (in mice) effects of BDQ and drug-drug interactions may have contributed to the lack of *in vivo* efficacy of the combination. Gupta *et al*. described accelerated bactericidal activity by addition of verapamil in one mouse strain while the activity in a different mouse strain was very modest [14].

Disruption of MmpL5 by transposon insertion leads to attenuated virulence in mice, at least in some cases [15]. Overexpression of MmpL5 did not lead to increased fitness *in vitro* or increased virulence *in vivo*, but it did not result in a loss of fitness either. This increases the likelihood that non-target based resistance will be used by mycobacteria as a stepping stone leading to higher level, target-based resistance. Induction of efflux pumps was shown to be the first step in a general pathway to resistance to azithromycin, eventually leading to high-level chromosomal-mutation-related resistance as ordered events in an “antibiotic resistance arrow of time” [16]. The frequency of target-based and non-target based mu”tations for BDQ is very similar *in vitro* [3], but the latter are more likely to emerge first *in vivo*, as efflux-based mutations generally lead to lower levels of resistance (2 to 16-fold higher MICs) than target based mutations (16-1000 fold higher MICs). Indeed so far only non-*atpE* mutations could be isolated from mice (3 isolates) and patients (6 isolates) with increased MICs for BDQ.

Hartkoorn *et al.* recently described the *in vitro* isolation of CFZ-resistant strains, with cross-resistance to bedaquiline [5]. Both CFZ and BDQ can therefore select for efflux-based resistance, leading to cross-resistance between the two drugs. The cross-resistance between BDQ and CFZ is of great concern because of its clinical implications. Our findings suggest that BDQ and CFZ will not protect each other against the emergence of resistance. In patients, it is crucially important to use the combination BDQ+CFZ only if protected from emergence of resistance by additional antibiotics, that are not a substrate of the MmpL5 efflux pump.

In mice, the combination BDQ+CFZ leads to significantly better bactericidal and sterilizing efficacy, despite the cross-resistance between the two drugs [17]. It is important to note here that the 3 mouse mutants described above were isolated from mice that had been treated with BDQ+CFZ in combination with either amikacin alone, or amikacin and levofloxacin for 4 months, and then relapsed 3 months later. No resistant strains could be isolated from mice treated for 6 months with the same combinations, suggesting residual activity of the BDQ+CFZ combination, and/or sterilizing activity of the companion drug(s) between months 4 and 6. The emergence of resistant strains late in the treatment course, and their subsequent elimination by continuation of treatment, underlines the importance of treatment compliance and the risk of premature discontinuation of therapy.

There is an additional reason why premature discontinuation of treatment creates a high risk for the emergence of resistance to both BDQ and CFZ. As both drugs have very long half-lives [18], [19] early discontinuation results in lingering low plasma levels of BDQ and CFZ, without protection of companion drugs with shorter terminal half-lives. Sub-therapeutic concentrations of some TB drugs produce tolerance in actively growing TB cultures [20], although current data do not suggest upregulation of the MmpL5 pump by BDQ [21]. Sequential use of BDQ and CFZ – or CFZ and BDQ may also increase the chance to develop efflux-based resistance.

Finally, our findings have implications for the development of rapid genotypic drug susceptibility tests (DST). To monitor for emergence of resistance to BDQ, both *Rv0678* and *atpE* genes of baseline and post-baseline isolates should be sequenced. SNP based assays are less likely to become the method of choice for rapid DST testing, as SNPs have already been described in many positions of both genes. A database linking phenotypic changes in MIC with genotypic changes will need to be generated to allow for clinical decisions based on genotypic DST data.

## Methods

### 1.1 Ethic statement

The animal study reported in this manuscript was carried out in compliance with the recommendations in the Guide for the Care and Use of Laboratory Animals of the National Institutes of Health. All animal procedures were approved by the Johnson & Johnson ethical committee for the use of animals. Humane endpoints were introduced to minimize animal pain and suffering.

### 1.2 Bacterial strains, media and growth conditions

Preclinical *in vitro* isolates EH 3.2, EH 3.3 and EH 3.6 originated from the MDR TB strain EH 3.0 and were isolated as previously described [3]. Preclinical *in vivo* isolates BCA4, BCA8 and BCLA2 were isolated from mice infected with H37Rv which were first treated for 4 months with a combination of 25 mg/kg BDQ, 20 mg/kg CFZ, and 150 mg/kg amikacin (BCA4 and BCA8 isolate) or BDQ, CFZ, amikacin and 300 mg/kg levofloxacin (BCLA2 isolate). After this 4 months treatment period, mice were kept for 3 additional months without any treatment before being sacrificed and assessed for relapses. Lung homogenates were plated on 7 H11 agar plates without BDQ and emerging colonies were tested for BDQ sensitivity.

Clinical isolates studied (listed in Table 2) came from patients participating in study C209 (NCT00910871). All available paired (baseline and post-baseline) samples with increased post-baseline BDQ MICs from this trial were studied.

### 1.3 Antimicrobial sensitivity testing

MICs were determined using two-fold dilution in 7 H9 medium with the resazurin microtitre assay (REMA) as previously described [22]. The MIC was interpreted as the lowest concentration that prevented a change in color from blue to purple or pink.

### 1.4 DNA manipulations and sequence analysis

#### 454 pyrosequencing

Genomic DNA from both the parent (EH 3.0) and two mutant (EH 3.2 and EH 3.6) strains was pyrosequenced using the GS FLX shotgun sequencing protocol from 454/Roche (GS-FLX, Roche Applied Science) following manufacturer’s instructions. Raw sequence data from the 454 sequencing experiments have been submitted to the Sequence Read Archive database (http://www.ncbi.nlm.nih.gov/Traces/sra/) with the accession numbers SRP042318, SRP042320 and SRP042321.

#### Genome assembly and variant detection

For each isolate, all individual sequences were assembled with high stringency onto the *M. tuberculosis* H37 Rv reference sequence (GenBank NC_000962), using CLCbio genomics workbench 6.0.1 (CLC, Denmark). Following read assembly, a consensus sequence (representing the genome sequence of the sample) was retrieved for each of the 3 samples. Next, both mutant genome sequences were aligned to the genome of the parent strain and positional differences (conflicts) were calculated using CLCbio. Resulting variants per mutant strain were compared with the parent strain. Each of the identified variants was checked individually in the original read assembly of the sample in order to distinguish genuine variants from errors introduced during the sequencing process or assembly.

#### PCR amplification and Sanger sequencing

A 649pb DNA fragment containing *Rv0678* gene and 143 bp upstream *Rv0678* was amplified by PCR using primers CV010 and CV017 (Table S3). In the case of EH 3.6, this PCR fragment is 2007 bp, so additional PCRs with primers Rv0678 Fwd 2, Rv0678 Rev 2, IS6110_AS F and IS6110_AS R were necessary for sequencing the product. The PCR products were gel purified using QIAquick Gel Extraction Kit (QIAGEN), and further sequenced with the same primers used for amplification. For analysis of the sequences, *Rv0678* sequence from *M. tuberculosis* H37Rv was taken as a reference [23] (http://tuberculist.epfl.ch).

### 1.5 Proteome analysis

Protein extraction and analysis were performed as previously described [21].

#### Sample preparation

*M. tuberculosis* strains were cultured in 7H9 broth to mid-log phase. Cell lysate was prepared in 2 ml lysis buffer and passed 3 times through the French Press at 14000 Psi in a small cell. Cell debris was removed by centrifugation and supernatant was collected.

#### Digestion and iTRAQ Reagent Labeling

The proteins were cleaned up by cold ethanol/acetone precipitation and digested with trypsin. After digestion, the peptides were labelled using iTRAQ labeling method. The samples were mixed and dried down using vacuum centrifugation.

#### Nano-LC MS/MS

Peptide mixtures were loaded on a HPLC Agilent 1200 system. After elution, they were further analysed by an EASY nano-HPLC system connected to an LTQ OrbiTrap XL mass spectrometer.

#### Data analysis

The raw MS/MS data were analysed using Proteome Discoverer v1.3 beta. MS/MS spectra were converted to.mgf files and searched against the NCBI database (taxonomy: *Mycobacterium tuberculosis* H37Rv). The protein ratios were calculated based on the peptide ratios of the individual proteins. The peptide ratios were log2 transformed and normalized against the median. To find regulated proteins, all outlier peptides were removed and the protein ratio recalculated. The standard deviation (σ) of the protein ratios were calculated after removing outliers. Every protein was accepted as regulated with a log ratio of +/−2*σ, (2*σ = 95.4% confidence interval).

### 1.6 Generation of overexpression strains

Replicative plasmids for overexpression of *mmpS5*, *mmpL5* or the operon *mmpS5-mmpL5* were cloned by GeneArt Gene Synthesis (Life Technologies). The three inserts, of 444 bp, 2910 bp and 3335 bp respectively, were synthesized *de novo* and cloned in pSD5 vector [10] to generate three independent plasmids (pCVGA26, pCVGA28 and pCVGA30, Table 6). The restriction sites *Nde*I and *Mlu*I of pSD5 vector were used for cloning the inserts under control of Hsp65 strong promoter. These plasmids and the control pSD5 vector were subsequently transformed in *M. tuberculosis* H37Rv to generate the strains overexpressing MmpS5 (strain H37Rv pCVGA26), MmpL5 (strain H37Rv pCVGA28), MmpS5-MmpL5 (strain H37Rv pCVGA30) and the control strain H37Rv pSD5. The presence of the corresponding plasmids was verified by PCR using the oligonucleotides described in (Table S3).

### 1.7 Fitness and virulence of resistant strains

#### *In vitro* competition assay

*M. tuberculosis* wild-type strain H37Rv, and the BDQ resistant mutants BCLA2 and BCA4 were used for growth competition testing *in vitro*. The cultures were grown to late logarithmic phase, adjusted to an OD_600nm_ of 0, 1 and subsequently used for inoculating 2 independent flasks containing 20 ml of 7H9/0,05% tween/OADC broth with i) 30 µl of H37Rv culture and 30 µl of BCLA2 cultures and ii) 30 µl of H37Rv and 30 µl of BCA4 cultures. The two flasks were incubated at 37°C. On days 0, 3, 5, 7, 10, 12 and 14, serial dilutions of both cultures were plated in duplicate on drug-free 7H11/OADC plates. In parallel, the same dilutions were plated in duplicate on 7H11/OADC plates containing BDQ at 0.08 µg/ml. The number of mutant (BCLA2 or BCA4) cells in the competition assay was determined from the number of colonies grown in BDQ-containing plates; the number of H37Rv cells in the competition assay was calculated from the number of colonies in the drug-free plates minus the number of mutant cells.

#### *In vivo* growth

To assess the effect of mutations in RV0678 on growth *in vivo*, groups of 8 mice were infected intravenously with 0.9–1.7×10^5^ CFU of wild-type H37Rv or the BDQ resistant (mouse) isolates BCLA2, BCA4 and BCA8. Mice were sacrificed and lung CFU titers determined at day 1 after inoculation, and weeks 2, 4 and 6.

### 1.8 *In vivo* effect of efflux pump inhibitors

To assess the effect of increased MICs on the efficacy of BDQ, 8 groups of 6 mice were infected intravenously with either wild-type H37Rv or the BDQ resistant (mouse) isolates BCLA2,BCA4 and BCA8. Treatment started at 12 days post infection with 6.25 mg/kg or 50 mg/kg of BDQ, in the absence or presence of verapamil dosed at 25 mg/kg. Both BDQ and verapamil were dosed orally, 5 times per week. After 4 weeks of treatments, mice were sacrificed and lung CFU titers determined. Differences were calculated versus week 2 baseline CFU counts as presented in Fig. S1B.

### 1.9 Statistical methods

To assess the contribution of verapamil to *in vivo* efficacy of BDQ, CFU counts of 8 groups of mice were evaluated using t-tests. P-values in all groups were >0.64 when correlated for multiple testing (Bonferoni correction).

## Supporting Information

Figure S1 Fitness of *Rv0678* mutants. **A.** *In vitro* competition assays between BCLA2 and BCA4 strains and their parent strain H37Rv. Mixed cultures of mutant and parent strains were grown in 7H9 broth and plated on days 0, 3, 5, 7, 10, 12 and 14, in parallel on selective and non-selective 7H10 agar plates. The left y axis depicts the growth of the mutant and parent strains as the total numbers of CFU/ml. The right y axis depicts the ratio of log numbers of CFU of resistant mutants (M)/log numbers of CFU of susceptible parent (H37Rv). **B.** Growth curves of H37Rv-derived *Rv0678* mutants in vivo. Groups of 8 mice were infected intravenously with either wild-type H37Rv or the BDQ resistant isolates BCLA2 and BCA4 and BCA8. One group was sacrificed after one day, 2, 4 and 6 weeks to determine lung CFUs. DL = detection limit.
(TIF)

Figure S2 Replicative plasmids derived from pSD5 used for overexpressing either *mmpS5*, *mmpL5* or the complete operon *mmpS5-mmpL5* in *M. tuberculosis* H37Rv. The inserts were cloned in the restriction sites *Nde*I and *Mlu*I, under control of the strong promoter P_Hsp65_.
(TIF)

Table S1 Regulation of the proteome of EH 3.2 and EH 3.6 compared with the parent strain 3.0. The relatively quantified expression of the complete dataset (1455 proteins) is shown. Proteins lacking the Rv number in the list could not be found in *M. tuberculosis* H37Rv strain, and are referred to other mycobacterial species.
(XLS)

Table S2 Top 9 downregulated proteins of the BDQ-resistant strains EH 3.6 and EH 3.2 compared to EH 3.0. Proteins displaying the highest differential downregulation factors (top 9 proteins) are indicated.
(DOC)

Table S3 Oligonucleotides used in this study.
(DOC)
