# Supplementary figures and images for: Acquired Resistance of Mycobacterium tuberculosis to Bedaquiline
Source: PLoS One. 2014 Jul 10;9(7):e102135. doi: 10.1371/journal.pone.0102135 (PMC4092087; doi:10.1371/journal.pone.0102135)

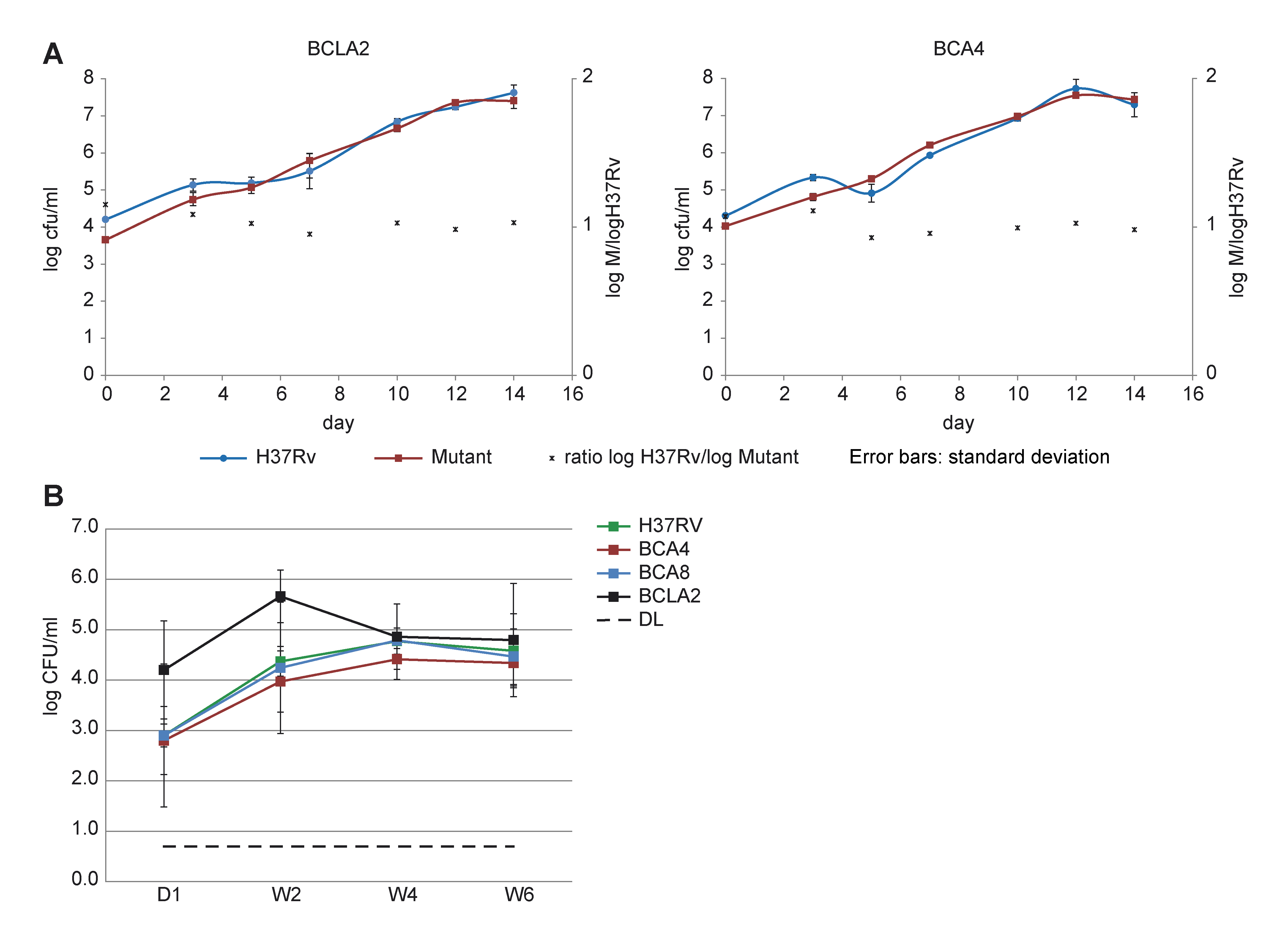

Supplement: Figure S1 — Fitness of Rv0678 mutants. A. In vitro competition assays between BCLA2 and BCA4 strains and their parent strain H37Rv. Mixed cultures of mutant and parent strains were grown in 7H9 broth and plated on days 0, 3, 5, 7, 10, 12 and 14, in parallel on selective and non-selective 7H10 agar plates. The left y axis depicts the growth of the mutant and parent strains as the total numbers of CFU/ml. The right y axis depicts the ratio of log numbers of CFU of resistant mutants (M)/log numbers of CFU of susceptible parent (H37Rv). B. Growth curves of H37Rv-derived Rv0678 mutants in vivo. Groups of 8 mice were infected intravenously with either wild-type H37Rv or the BDQ resistant isolates BCLA2 and BCA4 and BCA8. One group was sacrificed after one day, 2, 4 and 6 weeks to determine lung CFUs. DL = detection limit. (TIF) [file pone.0102135.s001.tif]

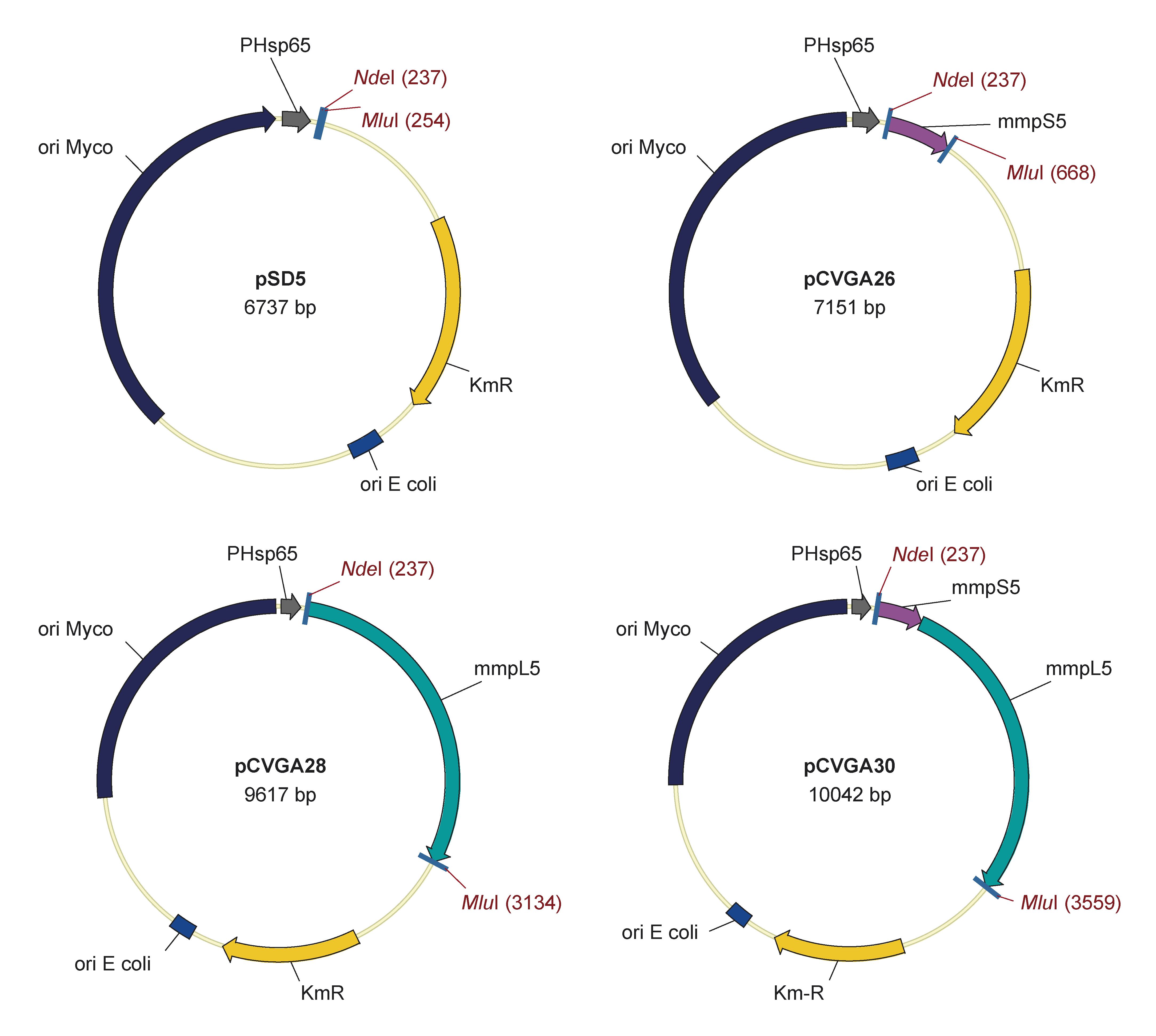

Supplement: Figure S2 — Replicative plasmids derived from pSD5 used for overexpressing either mmpS5, mmpL5 or the complete operon mmpS5-mmpL5 in M. tuberculosis H37Rv. The inserts were cloned in the restriction sites NdeI and MluI, under control of the strong promoter PHsp65. (TIF) [file pone.0102135.s002.tif]
