# Supplementary material for: Acquired Resistance of Mycobacterium tuberculosis to Bedaquiline
Source: PLoS One. 2014 Jul 10;9(7):e102135. doi: 10.1371/journal.pone.0102135 (PMC4092087; doi:10.1371/journal.pone.0102135)
Supplement: Table S2 — Top 9 downregulated proteins of the BDQ-resistant strains EH 3.6 and EH 3.2 compared to EH 3.0. Proteins displaying the highest differential downregulation factors (top 9 proteins) are indicated. (DOC) [file pone.0102135.s004.doc]

**Supplementary Table S2**

| **Rv number** | **gene** | **Description** | **Fold change EH3.6/EH3.0** | **Fold change EH3.2/EH3.0** |
| --- | --- | --- | --- | --- |
| Rv0858c | *dapC* | Probable N-succinyldiaminopimelate aminotransferase DapC (DAP-at) | 0.7 | 0.7 |
| Rv0830 | *Rv0830* | Possible S-adenosylmethionine-dependent methyltransferase | 0.7 | 0.7 |
| Rv2982c | *gpdA2* | Probable glycerol-3-phosphate dehydrogenase | 0.7 | 0.7 |
| Rv3843c | *Rv3843c* | Probable conserved transmembrane protein | 0.7 | 0.7 |
| Rv2860c | *glnA4* | Probable glutamine synthetase GlnA4 (glutamine synthase) | 0.7 | 0.7 |
| Rv2725c | *hflX* | Probable GTP-binding protein HflX | 0.7 | 0.7 |
| Rv2416c | *eis* | Enhanced intracellular survival protein Eis, GCN5-related N-acetyltransferase | 0.6 | 0.7 |
| Rv0966c | *Rv0966c* | Conserved protein | 0.5 | 0.5 |
| Rv3196A | *Rv3196A* | Unknown protein | 0.5 | 0.7 |

**Supplementary Table S2. Top 9 downregulated proteins of the BDQ-resistant strains EH 3.6 and EH 3.2 compared to EH 3.0**. Proteins displaying the highest differential downregulation factors (top 9 proteins) are indicated.
