# Supplementary material for: Acquired Resistance of Mycobacterium tuberculosis to Bedaquiline
Source: PLoS One. 2014 Jul 10;9(7):e102135. doi: 10.1371/journal.pone.0102135 (PMC4092087; doi:10.1371/journal.pone.0102135)
Supplement: Table S3 — Oligonucleotides used in this study. (DOC) [file pone.0102135.s005.doc]

**Supplementary Table S3**

| **Oligonucleotide** | **5'->3' Sequence** | **Reference** |
| --- | --- | --- |
| *For amplifying and sequencing* | | |
| CV010-MmpSL5-4 | ATGGCGACCACAACCAGG | This study |
| CV017-MmpSL5-10 | TTTTACGCGTGTTGCTCATCAGTCGTCCTC | This study |
| Rv0678 Fwd 2 | GCGGCTATTTCGAGTCCA | This study |
| Rv0678 Rev 2 | CTCATCAGTCGTCCTCTCC | This study |
| IS6110_AS F | TGGCGTTGAGCGTAGTAG | This study |
|  |  |  |
| IS6110_AS R | TCTTGGAAAGGATGGGGT | This study |
| *For verifying the overexpression strains* | | |
| CV018-pSD5F | GCGATATCCGGAGGAATCAC | This study |
| CV023-MmpL5-1 | GCGAGTTTCACCTGAACGTA | This study |
| CV028-pSD5R | GCTTGCAGGGAGTCAAAGG | This study |
| CV029-MmpL5-2 | GTGCTGATCTGGCAACACAT | This study |
| CV032-Km903F | GAGCCATATTCAACGGGAAA | This study |
| CV033-Km903R | CGAGCATCAAATGAAACTGC | This study |

**Supplementary Table S3. Oligonucleotides used in this study**.
